# Sustained low peritoneal effluent CCL18 levels are associated with preservation of peritoneal membrane function in peritoneal dialysis

**DOI:** 10.1371/journal.pone.0175835

**Published:** 2017-04-17

**Authors:** Marta Ossorio, María Auxiliadora Bajo, Gloria del Peso, Virginia Martínez, María Fernández, María José Castro, Aranzazu Rodríguez-Sanz, Rosario Madero, Teresa Bellón, Rafael Selgas

**Affiliations:** 1 La Paz University Hospital, Department of Nephrology, Hospital La Paz Health Research Institute-IdiPAZ, REDinREN, Fibroteam, Madrid, Spain; 2 Hospital La Paz Health Research Institute-IdiPAZ, Madrid, Spain; 3 La Paz University Hospital, Department of Statistics, Hospital La Paz Health Research Institute-IdiPAZ, Madrid, Spain; Seoul National University College of Pharmacy, REPUBLIC OF KOREA

## Abstract

Peritoneal membrane failure (PMF) and, ultimately, encapsulating peritoneal sclerosis (EPS) are the most serious peritoneal dialysis (PD) complications. Combining clinical and peritoneal transport data with the measurement of molecular biomarkers, such as the chemokine CCL18, would improve the complex diagnosis and management of PMF. We measured CCL18 levels in 43 patients’ effluent and serum at baseline and after 1, 2, and 3 years of PD treatment by retrospective longitudinal study, and evaluated their association with PMF/EPS development and peritoneal risk factors. To confirm the trends observed in the longitudinal study, a cross-sectional study was performed on 61 isolated samples from long-term (more than 3 years) patients treated with PD. We observed that the patients with no membrane dysfunction showed sustained low CCL18 levels in peritoneal effluent over time. An increase in CCL18 levels at any time was predictive of PMF development (final CCL18 increase over baseline, p = .014; and maximum CCL18 increase, p = .039). At year 3 of PD, CCL18 values in effluent under 3.15 ng/ml showed an 89.5% negative predictive value, and higher levels were associated with later PMF (odds ratio 4.3; 95% CI 0.90–20.89; p = .067). Moreover, CCL18 levels in effluent at year 3 of PD were independently associated with a risk of PMF development, adjusted for the classical (water and creatinine) peritoneal transport parameters. These trends were confirmed in a cross-sectional study of 61 long-term patients treated with PD. In conclusion, our study shows the diagnostic capacity of chemokine CCL18 levels in peritoneal effluent to predict PMF and suggests CCL18 as a new marker and mediator of this serious condition as well as a new potential therapeutic target.

## Introduction

Peritoneal dialysis (PD) is a technique for substitution of kidney function. Due to the use of a biological membrane with bioincompatible dialysis solutions the peritoneum suffers alterations that limit its use and long-term viability. This bioincompatibility ultimately leads to ultrafiltration failure (UFF), irreversible structural fibrosis, and potentially, encapsulating peritoneal sclerosis (EPS).

Clinical risk parameters and functional data of peritoneal membrane transport, such as high-fast small solute transport and lower free water transport, are conventionally used to predict functional and structural peritoneal membrane damage [1,2,3,4]. Although a continuum between peritoneal fibrosis and EPS has not been established, treatment for more than 4 years with bioincompatible PD solutions is known to present the highest risk for EPS [2,5]. Episodes of peritonitis [1,2,6], glucose degradation products (GDPs) [1,7], epithelial to mesenchymal transition (EMT) of mesothelial cells [8], and time are factors promoting peritoneal dysfunction.

Early clinical signs of peritoneal transport alterations have not been sufficient to establish scientifically guided evidence for early EPS diagnosis [2]. The possibility of combining this approach with molecular biomarkers would improve the detection and prevention of peritoneal functional and structural failure. The peritoneal membrane failure has been associated with peritoneal membrane fibrosis and EMT [9]. Alternatively activated macrophages (M2) can contribute to the fibrotic process of the peritoneum under PD [10]. M2 macrophages have a specific phenotype, being the chemokine CCL18 one of the hallmarks of this subpopulation [11]. In addition to the generation of extracellular matrix [12] M2 can stimulate fibroblast proliferation via CCL18 [10, 11, 13]. Moreover, coculture of M2 with fibroblasts enhances the production of collagen and this is partly dependent on CCL18 [13,14]). CCL18 chemokine is produced by peritoneal M2 macrophages and is notably present at easily measurable levels in the effluent of patients treated with PD [10,15]. High plasma CCL18 levels have been involved in progressive fibrosing disorders such as pulmonary and liver fibrosis [14,16].

We found that high levels of CCL18 in peritoneal effluent were associated with UFF and with later development of EPS in a small number of patients [9]; similar data have been reported by other authors [15]. Our objective was to analyze the predictive capacity of CCL18 levels in serum and peritoneal effluent to herald peritoneal membrane damage, as shown by functional data [1], or the development of EPS. We performed a longitudinal retrospective study based on our collection of frozen peritoneal effluents and serum samples from patients treated with PD for more than 2 years. To confirm the trends observed in the longitudinal study, a transversal study was performed on 61 isolated samples from long-term patients treated with PD.

## Patients and methods

### Study participants

A retrospective longitudinal study was performed as intention-to-treat on 43 patients from the peritoneal dialysis unit of La Paz University Hospital (Madrid, Spain) between December 1999 and December 2010, who underwent more than 2 years of PD treatment. Demographic data are shown in S1 Table. Samples and clinical data were collected at 4 time points: baseline (defined as the first 6 months of PD), 1, 2, and 3 consecutive years.

The exclusion criteria were age under 18 years, solid or hematological tumor, viral hepatitis, acute liver disease, acute inflammatory processes, significant allergic affectation, pulmonary fibrosis or significant organ fibrosis, deposit disease, or significant connective-dermatologic pathology.

Peritoneal functional data from these patients are described in Table 1. It is noteworthy that 13 patients required a high daily peritoneal glucose load to remain in appropriate extracellular volume status at year 3, with average peritoneal functional data (Dialysate/Plasma of Cr [Cr D/P], Mass Transfer-Area Coefficient of creatinine [Cr-MTAC] and urea [U-MTAC]) stable over the follow-up. Peritoneal membrane failure (PMF) and EPS cases were diagnosed during or after the study time. Ten patients eventually developed PMF. The mean follow-up of the PMF group was 46.2 (range 22–61) months. Similarly, the mean follow-up of patients without PMF was 35.2 (range 13–65) months (nonsignificant). No demographic differences were found between the groups. Only 2 patients were diagnosed with EPS, at 80 and 91 months

**Table 1:** Peritoneal function and membrane failure risk factors during a 3-year follow-up of 43 patients treated with PD, followed longitudinally.

Peritoneal function	Baseline (N = 43)	1 year of PD (N = 43)	2 years of PD (N = 42)	3 years of PD (N = 35)
UREA MTAC ml/min; mean ± SD	24.6 ± 5.9	23.8 ± 5.5	24.1 ± 5.5	22.6 ± 5.2
Cr MTAC (ml/min) mean ± SD	9.2 ± 3.8	9 ± 2.7	9.6 ± 2.8	8.5 ± 3
Peritoneal protein losses (gr/24h) mean ± SD	5.9 ± 1.8	6.4 ± 2.1	6.5 ± 2	6.2 ± 2
Na Sieving (mean ± SD)	3.4 ± 2.2	4 ± 2.2	3.3 ± 2.9	4 ± 2.6
Cr D/P (mean ± SD)	0.71 ± 0.06	0.70 ± 0.07	0.69 ± 0.07	0.66 ± 0.08
RRF (ml/min) mean ± SD)	6.5 ± 2.4	4.3 ± 2.9	3.3 ± 3	2.5 ± 2.7
D/P Cr >0.8	3 (7.1%)	3 (7.1%)	5 (12.2%)	3 (9.1%)
Cr MTAC >12	5 (12.2%)	6 (14.6%)	8 (20.5%)	3 (10.3%)
Ultrafiltration failure (UFF)	6 (15.4%)	3 (7.3%)	2 (5%)	4 (13.8%)
Serum albumin (g/dL) (mean±SD)	3.15±0.28	3.05±0.33	3.09±0.33	3.05±0.34
Membrane failure risk factors	Baseline (n = 43pts)	1 year on PD (n = 43pts)	2 years of PD (n = 42pts)	3 years of PD (n = 35pts)
Peritonitis (n)	3 (7.1%)	8 (19%)	15 (36.6%)	13 (38.2%)
Days of accumulated inflammation	0.24	0.88	1.9	2.91
Hemoperitoneum	0	0	0	1
High Glucose (≥50% glucose 2.27%)	2 (4.9%)	7 (16.7%)	13 (31.7%)	13 (38.2%)
Biocompatible solutions	19 (45.2%)	21 (50%)	23 (56.1%)	20 (58.8%)
Anuric patients	1 (2.9%)	4 (11.4%)	9 (25.7%)	11 (34.4%)

Abbreviations: MTAC: mass transfer-area coefficient; RRF: residual renal function; D/P Cr: dialysate/plasma creatinine, UFF: ultrafiltration failure

Sixty-one long-term (more than 3 years) patients treated with PD were analyzed in a transversal cross-sectional study (S2 Table). The last sample available before the cessation of PD was chosen for the analysis. The mean sampling time was 57.7 (range 30.4–152.9) months. Peritoneal function and risk factors for PMF corresponding to these patients are shown in Table 2. Twenty-two patients developed PMF at a median time of 55.9 (range 19–124) months; of these, 6 developed EPS at a median time of 106.3 months’ treatment with PD (range 60–147 months).

**Table 2:** Peritoneal function and membrane failure risk factors in 61 long-term patients treated with PD.

Peritoneal function		Membrane failure risk factors	
UREA MTAC (ml/min) (mean ± SD)	21.3 ± 5.4	Peritonitis (n)	1.47
Cr MTAC (ml/min) (mean ± SD)	9.6 ± 3.7	Days of accumulated inflammation	4.2
Peritoneal protein losses (gr/24h) (mean ± SD)	5.8 ± 2.7	Hemoperitoneum	2 (3.6%)
Sodium Sieving (mean ± SD)	3.2 ± 3	High Glucose (≥50% glucose 2.27)	28 (51.9%)
Cr D/P (mean ± SD)	0.70 ± 0.03	Biocompatible solutions	34 (63.0%)
RRF (ml/min) (mean ± SD)	1.9 ± 2.5	Anuric patients	22 (50.0%)
D/P Cr > 0.8	13 (22.4%)	High risk (anuric &/o DM1 &/o high glucose)	32 (52.5%)
Cr MTAC >12 ml/min	12 (21.1%)	Ultrafiltration failure (UFF)	17 (29.8%)
Serum albumin (g/dL) (mean±SD)	2.89 ± 0.37		

Abbreviations: MTAC: mass transfer-area coefficient; RRF: residual renal function; D/P Cr: dialysate/plasma creatinine; DM1: diabetes mellitus type 1, UFF: ultrafiltration failure

The study was approved by the Research Ethics Committee of La Paz University Hospital (PI12_0024). All clinical investigations have been conducted according to the principles expressed in the declaration of Helsinki and written informed consent was obtained from all the patients.

### Peritoneal membrane function estimation: Peritoneal transport

Peritoneal transport was estimated by calculating small solute transport (urea [U] and creatinine [Cr] dialysate to plasma [D/P] and peritoneal mass transfer coefficients [MTAC]) under a 3.86%–4.25% glucose exchange with a 4 h dwell time [17], performed routinely every 6–12 months. Ultrafiltration (UF) capacity was estimated by weighing the dialysis fluid prior to and after this dwell time. The serum and effluent samples were stored at -80°C after each peritoneal kinetic study. Residual renal function (RRF) was calculated as the mean of urea and creatinine renal clearances.

### Definition of peritoneal membrane failure

We defined peritoneal membrane failure (PMF) as Cr MTAC values over 12 ml/min or Cr D/P over 0.8 (all representing high/fast transport) and/or UF capacity less than 400 ml/4 h, all appearing after 2 or more years of PD treatment, without a specific precipitating event [1].

### Definition of encapsulating peritoneal sclerosis

EPS was diagnosed by surgical or histological direct visualization, the presence of two major criteria (compatible image by tomography and ultrasonography), or one major and two minor criteria (symptoms of bowel pseudo- or obstruction, high peritoneal transport).

### ELISA CCL18 and PAI-1

CCL18 was quantified in serum and 4-hour dwell peritoneal effluents using a DuoSet ELISA Development System (R&D Systems Europe, UK), according to the manufacturer’s instructions. On average, 1/1000 dilution and 1/50 dilutions were analyzed in serum and effluent samples, respectively.

PAI-1 was quantified in undiluted effluent samples using a DuoSet ELISA Development System (R&D Systems Europe, UK), according to the manufacturer’s instructions.

### Statistical analysis

The statistical analysis was performed using SPSS-15 software (IBM, Inc., Chicago, Il, USA).

We performed a descriptive analysis of serum and effluent samples at each time point. Parametric or nonparametric tests were applied according to the distribution of the samples. The data of serum and effluent CCL18/PAI-1 levels in the longitudinal study were analyzed using a linear mixed-effects regression model (LMERM), considering time and the intercept as random effects.

The development of PMF or EPS in relationship to the evolution of effluent CCL18 concentrations was investigated by Kaplan-Meier analysis. A Cox hazard analysis was used to analyze the association between the behavior of CCL18 in peritoneal effluent and the subsequent development of PMF or EPS.

The diagnostic capacity of CCL18/PAI-1 in effluent at the third year of PD treatment for prediction of PMF was evaluated by using the receiver operating characteristic (ROC) curve. Youden’s index was used to choose the point at which sensitivity and specificity were simultaneously maximized as the optimal cut-off point.

To analyze the additional value of CCL18 in effluent we conducted a multivariate analysis adjusted for classical membrane failure parameters (Cr D/P, Cr- or U-MTAC and UFF).

## Results

### Serum CCL18 levels during longitudinal study

Serum CCL18 values during follow-up are shown in S3 Table and S1A Fig. The average serum CCL18 concentration was 151.59±72.06 ng/ml. High individual variability was detected and no significant changes were found over time using the LMERM. The serum CCL18 levels did not show any significant association with clinical features, the use of biocompatible solutions, peritonitis, abnormal peritoneal transport, or treatment with immunomodulatory agents. Also, no significant association was found between serum CCL18 levels and the development of EPS or PMF.

### Effluent CCL18 levels during longitudinal study

The CCL18 values in effluent are shown in S3 Table and S1B Fig, none demonstrating overall significant changes during the follow-up (LMERM). However, some clinical situations were identified in which the concentration of CCL18 tended to increase.

Effluent CCL18 levels were elevated in all samples analyzed from patients with diabetes type 1 (DM1) compared with patients without DM1, without association with serum CCL18 levels (data not shown). Initial features at baseline, such as high peritoneal glucose load (4.60 vs. 2.54 ng/ml; p = .027), peritonitis at first year (4.55 vs. 2.64 ng/ml; p = .038), and high transport (Cr-MTAC >12: 4.35 vs. 2.64 ng/ml, p = .019; and D/P Cr >0.8: 4.61 vs. 2.66 ng/ml, p = .035), were also associated with higher CCL18 levels in effluent.

### Patients with no membrane dysfunction showed sustained low levels of CCL18 in peritoneal effluent over time

An observational analysis of the raw data gathered from the 43 retrospective longitudinal study patients revealed a group of 11 patients whose CCL18 levels in effluent remained without relevant changes throughout the study and with CCL18 levels in effluent lower than the mean values obtained in each time point, and below or close to the mean CCL18 effluent values determined in patients with no PMF in a previous study [10]. Therefore, two groups were defined: group 1, comprising the 11 patients with stable low CCL18 values in effluent, and group 2 including the remaining 32 patients (including patients with initial values higher that the mean baseline values, and patients whose effluent CCL18 levels increased along the time under study) (S2 Fig). Demographic data are shown in S4 Table. Peritoneal functional data in both groups are shown in Table 3.

**Table 3:** Peritoneal function and PMF risk factors in patients included in group 1 and group 2.

**Peritoneal function**		**Baseline**	**1 year of PD**	**2 years of PD**	**3 years of PD**
**Cr D/P (mean ± SD)**	Group 1	0.67±0.05	0.69±0.06	0.67±0.08	0.63±0.09
	Group 2	0.72±0.06	0.70±0.07	0.70±0.06	0.67±0.08
**Cr MTAC (ml/min) mean ± SD**	Group 1	7.53±1.80	8.01±2.32	8.37±2.35	6.67±2.35
	Group 2	9.83±3.70	9.48±2.75	10.08±2.75	9.2±2.9*
**UREA MTAC ml/min; mean ± SD**	Group 1	22.67±4.10	23.19±4.22	21.91±4.48	21.13±3.15
	Group 2	25.38±6.38	24.12±6.00	25.01±5.72	23.20±5.80
**Na Sieving (mean ± SD)**	Group 1	4.81±1.84	4.29±1.43	4.79±1.25	4.16±1.90
	Group 2	3.04±2.71	3.95±2.47	2.83±3.19	4.04±2.89
**Peritoneal protein losses (gr/24h) mean ± SD**	Group 1	6.02±1.75	6.69±2.48	6.36±2.22	5.80±2.44
	Group 2	5.88±1.89	6.35±2.16	6.65±2.06	6.48±1.84
**RRF (ml/min) mean±SD**	Group 1	6.27±2.11	3.88±3.33	2.64±2.57	2.43±2.9
	Group 2	6.60±2.56	4.55±2.89	3.59±3.18	2.59±2.67
**D/P cr >0.8**	Group 1	0	1 (9.1%)	1 (9.1%)	1 (11.1%)
	Group 2	2 (6.5%)	2 (6.5%)	4 (13.3%)	2 (8.,3%)
**MTC Cr >12ml/min**	Group 1	0	1 (9.1%)	0	0
	Group 2	2 (6.5%)	2 (6.5%)	5 (16.7%)	3 (14.3%)
**UFF**	Group 1	0	2 (22.2%)	0	2 (22.2%)
	Group 2	6 (20.7%)	3 (10%)	4 (19%)	2 (9.5%)
**Serum albumin (g/dL) (mean±SD)**	Group 1	3.15±0.28	3.17±0.29	3.15±0.37	3.12±0.31
	Group 2	3.15±0.28	3.01±0.34	3.07±0.32	3.02±0.35
**Membrane failure risk factors**		**Baseline**	**1 year of PD**	**2 years of PD**	**3 years of PD**
**Anuric patients**	Group 1	0	2 (22.2%)	3 (27.3%)	3 (27.3%)
	Group 2	1 (3.8%)	2 (7.7%)	6 (23.1%)	8 (34.8%)
**High glucose (≥50% glucose 2.27%)**	Group 1	0	0	3 (27.3%)	5 (55.6%)
	Group 2	2 (6.5%)	5 (18.8%)	10 (33.3%)	8 (32%)
**Biocompatible solutions**	Group 1	4 (26.4%)	5 (45.5%)	5 (45.5%)	4 (44.4%)
	Group 2	15 (48.4%)	16 (51.6%)	16 (51.6%)	18 (60%)
**Peritonitis (n)**	Group 1	0	2 (18.2%)	5 (45.5%)	4 (44.4%)
	Group 2	3 (9.7%)	6 (19.4%)	10 (33.3%)	9 (36%)
**Days of accumulated inflammation**	Group 1	0	0.73	1.55	2.89
	Group 2	0.32*	0.94	2.03	2.92

*p = 0.04 (Mann-Witney U test)

Abbreviations: MTAC: mass transfer-area coefficient; RRF: residual renal function; D/P Cr: dialysate/plasma creatinine; DM1: diabetes mellitus type 1, UFF: ultrafiltration failure

**Fig 1:**
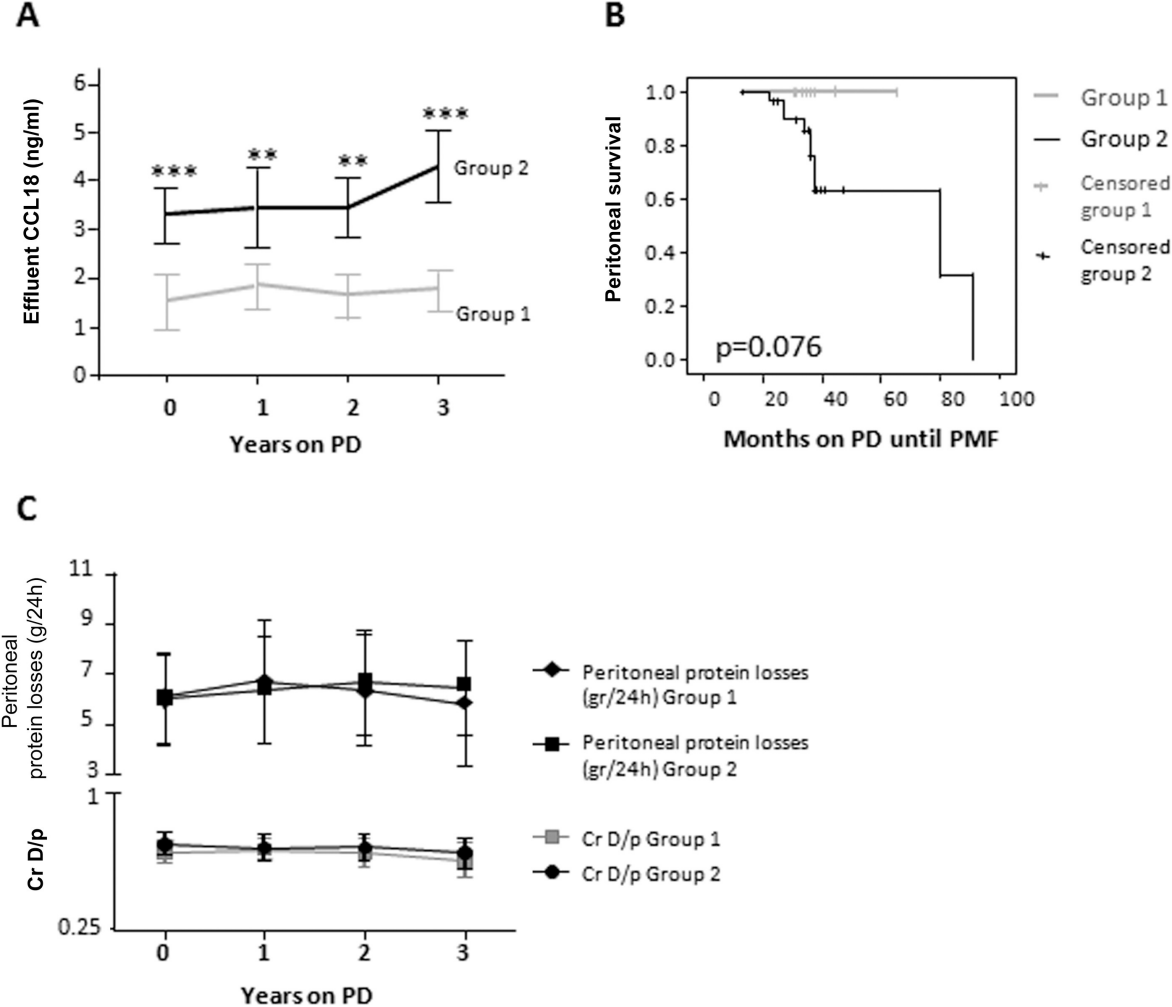
Low effluent CCL18 concentrations over time are found in patients with no peritoneal membrane failure. **(A)** Time course of effluent CCL18 concentrations define two groups of patients on PD. Eleven patients were included in Group 1, and 32 patients were included in group 2. Mean ± SD values are shown. Higher CCL18 effluent values were found in group 2 at all time points **p < .005; ***p < .001 (Mann-Whitney U test). **(B)** Kaplan-Meier analysis of PMF development in the patients included in group 1 and group 2. Patients with no sample at a given time point were censored. Log rank test p = .076. **(C)** Mean Cr D/P and peritoneal protein losses in patients included in Group 1 and Group 2 evaluated at different time points.

The differences in the concentration of CCL18 in peritoneal effluent between both groups were significant throughout the study (Fig 1A). A Kaplan-Meier analysis showed that no patient within group 1 (low and stable CCL18 levels in effluent) developed PMF within the 3 years, whereas patients with higher or growing levels of CCL18 in effluent eventually did (Fig 1B).

The elevated CCL18 effluent levels observed in group 2 were not associated with an increased peritoneal membrane transport of small solutes or proteins (Fig 1C).

### An increase in CCL18 in effluent heralds peritoneal membrane dysfunction

**Fig 2:**
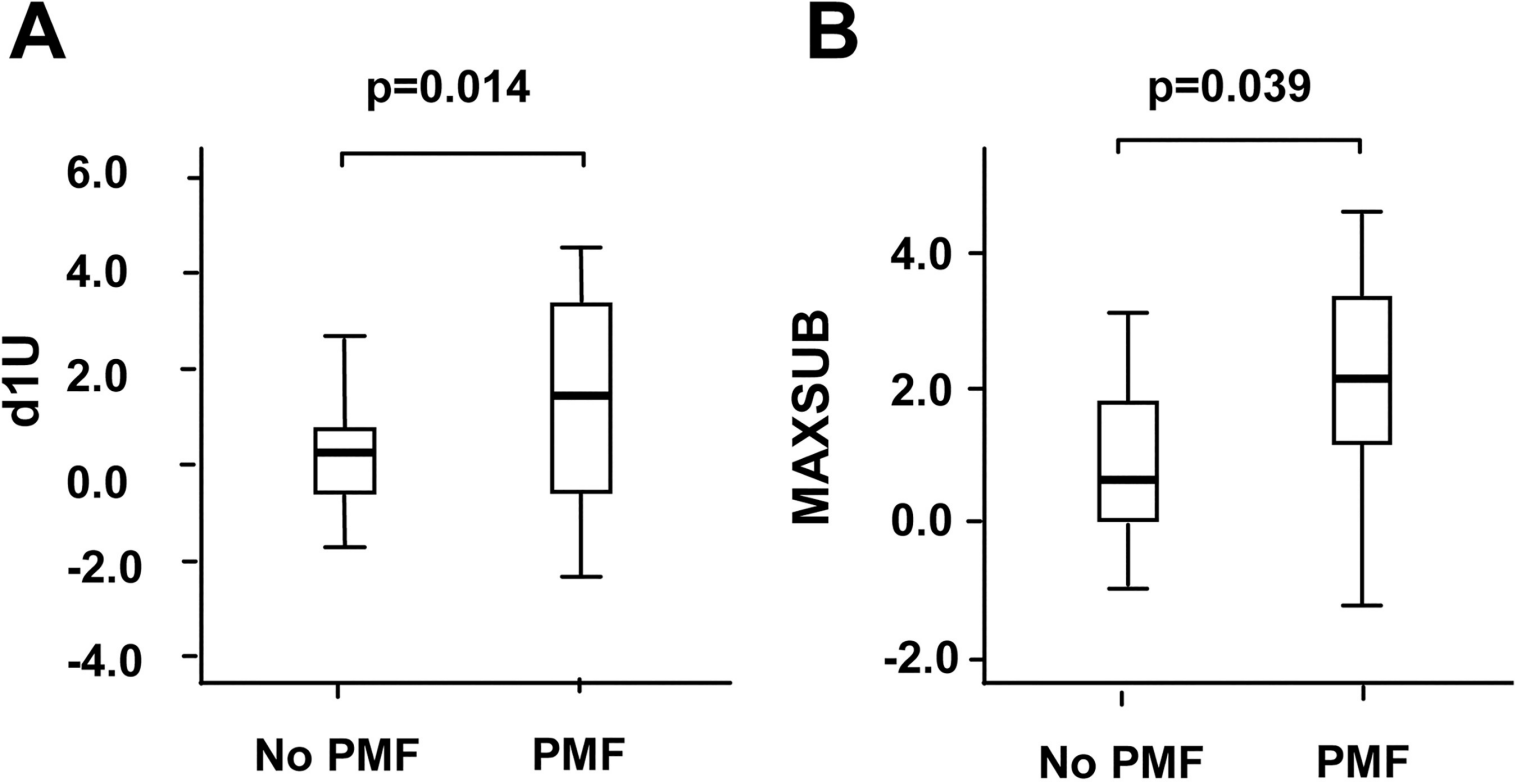
An increase in effluent CCL18 concentration heralds peritoneal membrane dysfunction. **(A)** The variable d1U was defined as the last CCL18 effluent value minus the baseline level in each patient included in the longitudinal analysis. **(B)** The variable Maxub was defined as the maximum minus the minimum CCL18 effluent concentration measured in each patient. A Cox hazard analysis of differences was performed on the patients who developed PMF (N = 10) and the patients who did not (N = 33).

Given we observed that sustained low CCL18 effluent concentrations appeared to be associated with improved peritoneal membrane survival, we explored whether an increase in CCL18 effluent levels over time on PD was associated with subsequent development of PMF. We defined two variables: 1) d1U CCL18 (the last CCL18 value in effluent minus the baseline level in each patient); and 2) Maxsub CCL18 (maximum CCL18 level in effluent during the follow-up minus the minimum CCL18 level measured in each patient). A Cox hazard analysis of these variables revealed that an increase in CCL18 in effluent at any time during the follow-up was significantly associated with the ultimate development of PMF (D1U CCL18, p = .014; and Maxsub CCL18, p = .039) (Fig 2).

### Patients who developed peritoneal membrane failure showed higher effluent levels of CCL18

**Fig 3:**
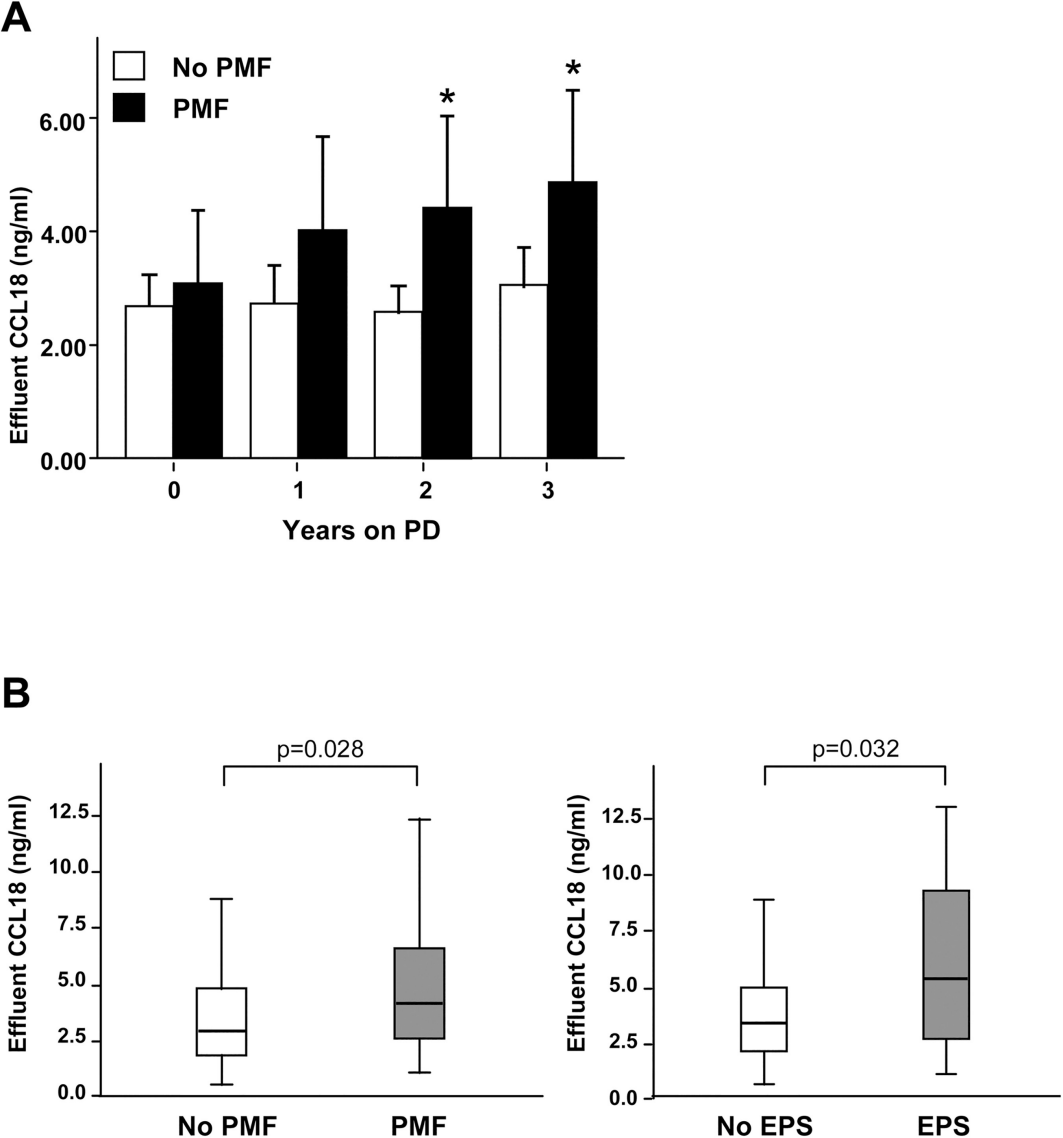
Patients who developed PMF showed higher CCL18 effluent levels. **(A)** Time course analysis of effluent CCL18 values in the patients who developed PMF (black bars; N = 10) or who did not (white bars; N = 33). Mean±SD are shown *: p < .05 (Mann-Whitney U test). **(B)** Cross-sectional study of 61 patients treated with PD with late (>3 years) peritoneal samples. Significantly higher CCL18 values were found in peritoneal effluent samples from the patients who developed PMF (N = 22) or EPS (N = 6) (Student’s t-test).

Higher concentrations of CCL18 were found during the entire follow-up period in effluents from the 10 patients who eventually developed PMF, with significant differences in the second year (4.23 vs. 2.68 ng/ml; p = .024) and third year (4.70 vs. 2.92 ng/ml; p = .006) of PD treatment, relative to patients without PMF (Fig 3A).

It is of note that the 2 patients included in the longitudinal study who finally developed EPS also showed higher CCL18 in effluent at baseline and during the first and second years of PD treatment, compared with the rest of the patients (data not shown). In addition, in the cross-sectional study, we observed significantly higher concentrations of CCL18 in peritoneal effluent from the 22 patients who developed PMF and in the 6 patients from this group who ultimately developed EPS (Fig 3B).

### Diagnostic capacity of CCL18 levels in effluent

**Fig 4:**
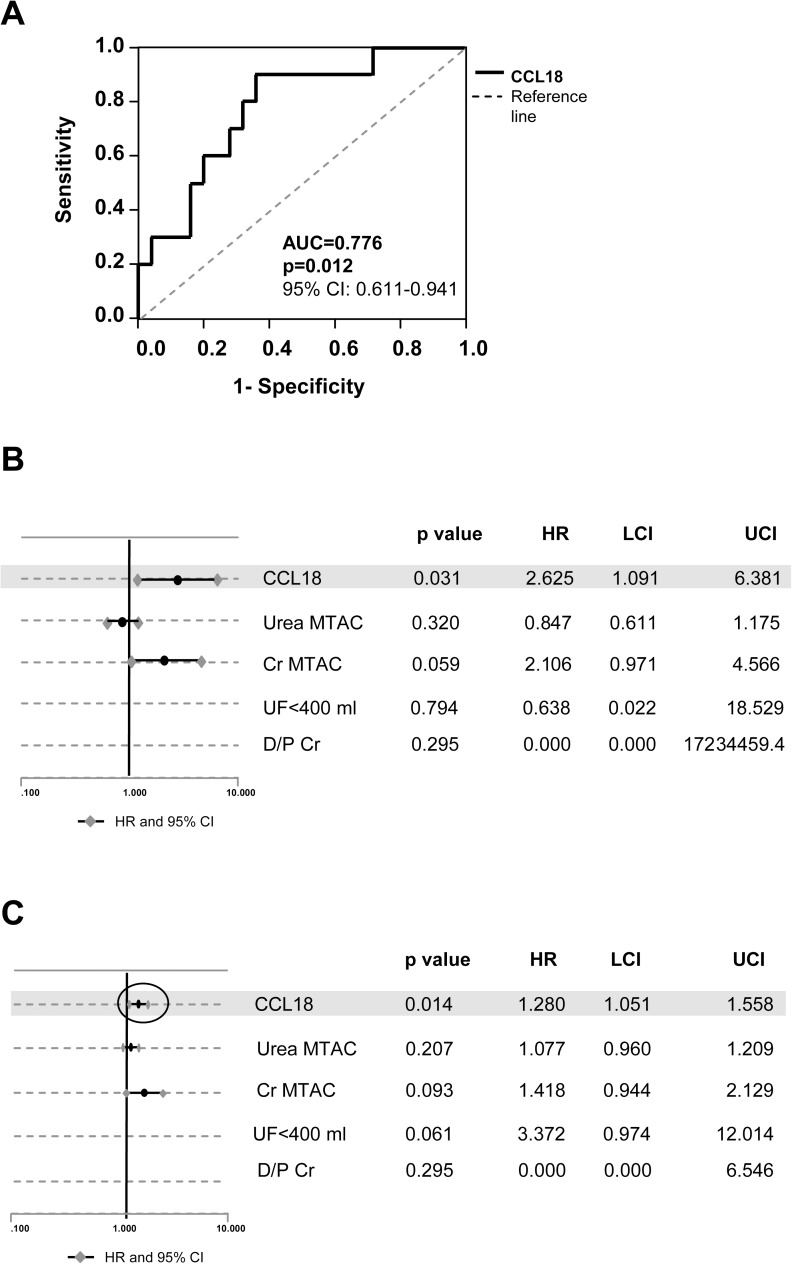
Effluent CCL18 concentrations predict the survival of the peritoneal membrane independent of classical transport parameters. **(A)** Receiver operating characteristic (ROC) curve analysis of CCL18 effluent concentrations in samples from patients at 3 years of PD treatment who did or did not develop PMF. (AUC: area under the curve; CI: confidence interval). **(B)** Forest plot showing the hazard ratio (HR) and 95% upper and lower confidence intervals (UCI and LCI) of CCL18 and peritoneal membrane transport parameters in 35 patients measured at the third year of PD treatment. CCL18 showed significant association with PMF independent of U-MTAC, Cr -MTAC, or D/P Cr. **(C)** Forest plot showing the HR and 95% UCI and LCI of CCL18 and peritoneal membrane transport parameters measured at the last effluent determination in 61 patients who were treated for more than 3 years with PD. CCL18 showed a significant association with PMF, independent of U-MTAC, Cr-MTAC, or D/P Cr.

A ROC curve was built to explore the diagnostic capacity of CCL18 in effluent at the third year of PD treatment to identify patients at PMF risk. The area under the curve was 0.776 (95% CI 0.611–0.941). The optimal CCL18 value in effluent that simultaneously maximized sensitivity (80%) and specificity (68%) to predict the subsequent development of PMF was 3.15 ng/ml or higher at year 3, with an 89.5% negative predictive value (NPV) (Fig 4A).

Using Cox regression analysis, we also observed an association between levels of CCL18 in effluent higher than 3.15 ng/ml at year 3 and a late diagnosis of PMF/EPS (OR 4.33, 95% CI 0.90–20.89; p = .067).

Finally, using a multivariate analysis we observed that levels of CCL18 in effluent at year 3 were independently associated with a risk of development of PMF/EPS adjusted for the classical peritoneal transport parameters (Cr-MTAC, Cr D/P, U-MTAC, UF), which were the best predictors of this outcome (Fig 4B). These results were confirmed in the cross-sectional study on long-term patients (Fig 4C).

### Analysis of PAI-1 effluent levels

It has been recently reported that effluent PAI-1 may help in monitoring peritoneal fibrosis [18]. Effluent levels of PAI-1 were evaluated in order to compare the predictive ability of this soluble factor with CCL18. In the longitudinal study, we observed a positive correlation between PAI-1 and CCL18 effluent levels at baseline (r = .31; p = .04) and at year 2 on PD (r = .42; p<0.001). However, this correlation was not found in the samples at years 1 and 3 of PD. Nonetheless, in the cross-sectional study CCL18 and PAI-1 effluent levels seemed to be related (r = .34; p<0.001).

**Fig 5:**
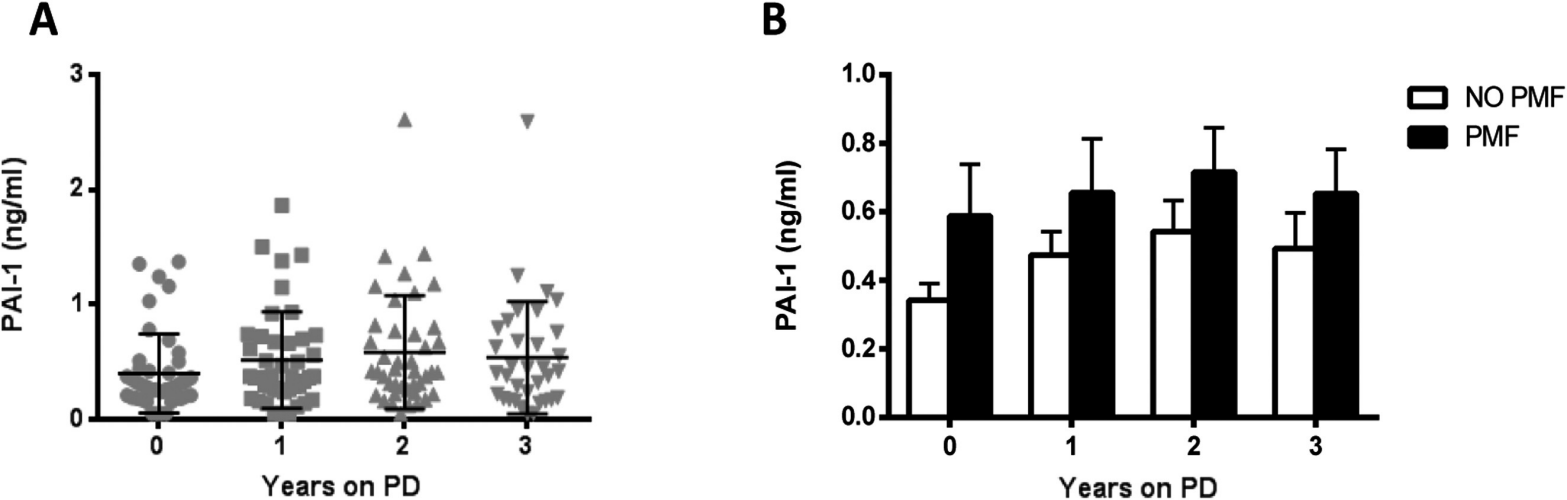
Time course analysis of PAI-1 values in patients treated with PD. **(A)** Scatter plots showing mean, and SD values **(B)** Effluent PAI-1 values in patients who developed PMF (black bars; N = 10) or who did not (white bars; N = 33) Mean ±SD are shown. No significant differences were detected at any time point (Mann-Witney U test)

The values obtained in the longitudinal study are shown in Fig 5A. No significant differences were detected at different time points. Similar to CCL18, higher effluent levels of PAI-1 were found on average at all time points in the longitudinal study in patients who developed PMF; although the differences were not statistically significant (Fig 5B).

**Fig 6:**
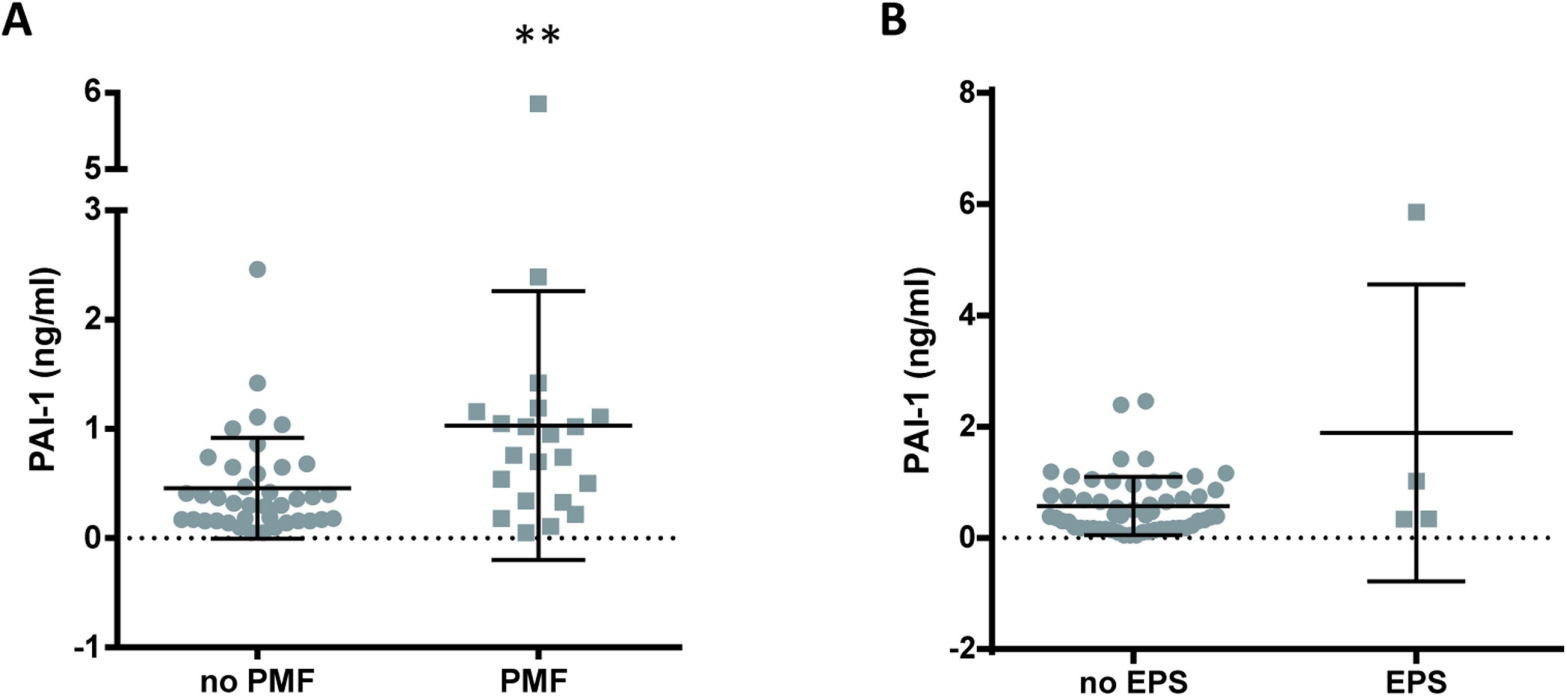
Cross-sectional study of PAI-1 values in 61 patients treated with PD with late (>3 years) peritoneal samples. Scatter plots showing mean, and SD values (A) Significantly higher levels were found in effluent samples from the patients who developed PMF (p = .0038; Mann-Witney U test). (B) PAI-1 effluent concentrations in patients who developed EPS. No significant differences were detected (Mann-Witney U test)

In the cross-sectional study significantly higher levels of PAI-1 were found in patients who developed PMF (1.03 *vs* 0.45 ng/ml; p = .0038) (Fig 6A). A trend to higher effluent values were found also in patients who developed EPS; although the differences were not statistically significant (Fig 6B).

Time-specific ROC curves were built. Effluent concentrations of PAI-1 at the third year of PD treatment showed lower capacity than CCL18 concentrations to predict PMF (AUC: 0.656; 95% CI: 0.41–0.9); not significant). Baseline concentrations of PAI-1 were evaluated as well showing also lower diagnostic power as compared to CCL18 effluent levels at year 3 of PD. The combination of CCL18 and PAI-1 did not improve the diagnostic power, likely because the high quantitative differences in the concentrations of both factors, cause a small contribution of PAI-1 to the variable; although it is possible also that at that time CCL18 is more relevant as predictor of peritoneal survival Effluent appearance rates were also evaluated for comparative purposes showing no improvement in the diagnostic capacity of CCL18 or PAI-1 (Table 4).

**Table 4:** ROC curve analysis of PAI-1 and CCL18 effluent concentrations and appearance rates (calculated as concentration x drained volume in 4h) at baseline (time 0) and after 3 years of PD treatment as diagnostic factors for PMF.

Concentration	Time	AUC (95% CI)	p value
PAI-1	Baseline	0.670 (0.44–0.89)	ns
	2 years	0.670 (0.47–0.86)	ns
	3 years	0.656 (0.41–0.90)	ns
**CCL18**	Baseline	0.602 (0.38–0.82)	ns
	2 years	0.681 (0.49–0.86)	ns
	**3 years**	**0.776 (0.61–0.94)**	**.012**
**CCL18+PAI-1**	Baseline	0.644 (0.42–0.86)	ns
	2 years	0.700 (0.51–0.88)	ns
	**3 years**	**0.773 (0.60–0.94)**	**.016**
**Appearance rates**			
PAI-1	Baseline	0.601 (0.32–0.87)	ns
	2 years	0.634 (0.42–0.84)	ns
	3 years	0.591 (0.30–0.87)	ns
CCL18	Baseline	0.575 (0.33–0.81)	ns
	2 years	0.641 (0.43–0.85)	ns
	3 years	0.776 (0.50–0.93)	ns

ns: Not significant

## Discussion

In this study, we demonstrated the diagnostic capacity of the chemokine CCL18 evaluated in peritoneal effluent to predict dysfunction of the peritoneal membrane under PD. The most remarkable result was that sustained low peritoneal CCL18 concentrations appeared among patients with no membrane dysfunction at the medium term. In contrast, patients who developed PMF showed higher or progressively increased CCL18 effluent levels. The increment observed in CCL18 effluent levels was not observed in CCL18 serum levels and was not associated with an increase in peritoneal membrane small solutes or protein transport (Cr D/P, Cr-MTAC, U-MTAC, peritoneal protein losses). This suggests a major local production of CCL18 in the peritoneum. At year 3 of PD treatment, CCL18 levels above 3.15 ng/ml accurately predicted ultimate membrane dysfunction (89.5% of negative predictive value), independent of peritoneal transport parameters (Cr-MTAC, Cr D/P, U-MTAC, UFF) at that time. To date, these parameters have been considered the primary criteria with a capacity to predict worsening of the peritoneal membrane’s functional status [2]. These criteria are now amended by our findings and by other reports demonstrating that specific biomarkers related to inflammation or repair processes, such as interleukin-6 and plasminogen activator inhibitor (PAI)-1, precede severe changes in peritoneal function [18,19].

However, contrary to other conditions such idiopathic pulmonary fibrosis [20], serum levels of CCL18 were not associated with peritoneal membrane status prediction.

Peritoneal functional changes such as UFF with high-fast small solute transport and lower free water transport have been shown to herald EPS [1,4,21]. This disorder is based on denser collagen at the peritoneal interstitium [21], limiting interstitial water circulation with preservation of AQP1 in the peritoneal capillary endothelium, the only factor related to date with free water transport in the peritoneum. Recently, isolated biomarker approaches such as the evaluation of PAI-1 in peritoneal effluent have been successful in predicting EPS [18]. The mechanism suggested for this marker/mediator is enhanced fibrin deposition and collagen secretion stimulated by PAI-1 in mesothelial cells. To investigate the power of CCL18 as a biomarker indicating the status of the peritoneal membrane, we defined PMF as the combined values of high solute transport and deficient UF capacity, with EPS being the extreme condition. The relationship between CCL18 in effluent and PMF was investigated in two groups of patients, including a first group of patients from whom serial samples were available (longitudinal study) and a second group of patients who were undergoing long-term PD treatment and from whom late samples were available (cross-sectional study).

The primary finding of the longitudinal study was from a subset of patients who maintained low and stable CCL18 levels in effluent throughout PD treatment, given none of these patients developed PMF during the follow-up period. We also found that an increase in CCL18 in effluent at any time was predictive of PMF development. Moreover, effluent levels of CCL18 at the second and third year of PD treatment were significantly higher in the patients who developed PMF. In the cross-sectional study, we also confirmed significantly higher effluent concentrations of CCL18 in the patients who ultimately developed PMF and/or EPS, in agreement with previous findings [10,15]. Because it is probably not feasible to evaluate CCL18 periodically in these patients, a ROC curve was used to analyze the predictive value of effluent CCL18 for development of PMF in samples from patients at the third year of PD treatment. CCL18 concentrations above 3.15 ng/ml were found to predict the subsequent development of PMF with 80% sensitivity and 89.5% NPV. We have also evaluated the capacity of effluent concentrations of PAI-1 to predict the development of PMF at different time points. The results revealed that in our patients, effluent CCL18 concentrations after 3 years of PD treatment are a better tool to predict the development of PMF.

Furthermore, CCL18 values were independent of the classic transport parameters (Cr-MTAC, Cr D/P, U-MTAC, UFF) admitted as the best predictors for this outcome [2,17].

From this point of view, any factor able to increase CCL18 at any time would be a risk factor for the development of PMF. It is of note that the 2 patients with DM1 showed higher CCL18 values in effluent not only during the later years on PD but also at early time points. Other early events, such as peritonitis or use of high glucose dialysis solutions, were also associated with higher CCL18 values (data not shown).

Furthermore, the fact that a group of patients with sustained low CCL18 in effluent did not develop peritoneal membrane dysfunction during the follow-up suggests that CCL18 is not only a biomarker, but it can also be involved in the cellular and molecular mechanisms leading to PMF. Previous studies suggest that PMF is related to fibrotic processes of the peritoneal membrane [22], and CCL18 has been previously related to fibrosis in various diseases [20,23–25]. In addition, CCL18 is able to promote the secretion of collagen [26] and the proliferation of fibroblasts [10]. Peritoneal M2 macrophages appear to be the primary source of CCL18 in patients treated with PD [10]. Factors able to bias macrophage activation toward an M2 phenotype are known to increase the expression and secretion of CCL18 [11]. Recently, CD163+ M2 macrophages have been demonstrated as one of the dominant cell populations in EPS peritoneal biopsies [27]. In line with this finding, peritoneal CD163+ macrophages secrete high quantities of CCL18 [10].

All these factors could support the “two-hit” theory that attempts to explain the etiology of simple peritoneal fibrosis leading to EPS, and M2 macrophages could be decisive in the second hit. Therapeutic interventions able to shift macrophage polarization from the M2 to the M1 phenotype could be of potential interest to preserve the peritoneal membrane throughout PD, thus preventing EPS.

Our study has limitations, such as the small sample size. Nonetheless, the marker behaved coherently throughout the various statistical explorations.

In conclusion, we have demonstrated the diagnostic capacity of CCL18 in peritoneal effluent to predict dysfunction of the peritoneal membrane. The most remarkable result is that sustained low CCL18 concentrations appear to guarantee no membrane dysfunction at the medium-term. In contrast, patients with higher or progressively increasing effluent CCL18 levels will develop PMF. At year 3 of PD treatment, CCL18 concentrations over 3.15 ng/ml predicted ultimate membrane dysfunction, independent of concurrent peritoneal transport parameters.

## Supporting information

S1 Table Demographic characteristics of 43 patients included in the longitudinal study. (PDF)

S2 Table Demographic characteristics of 61 patients included in the cross-sectional study. (PDF)

S3 Table Effluent and serum CCL18 levels during a 3-year follow-up of longitudinal study. (PDF)

S4 Table Demographic characteristics of patients from the longitudinal study included in Group 1 and Group 2. (PDF)

S1 Fig Longitudinal study of CCL18 values in patients treated with PD. Time course analysis of serum **(A)** and effluent **(B)** CCL18 values in 43 patients treated with PD during a 3-year follow-up period. Box plots show median, interquartile ranges, and SD. No significant changes were found overall throughout PD (ANOVA test).
(PDF)

S2 Fig **Effluent concentrations of CCL18 in patients included in group 1 and group 2 (A)** Group 1(orange symbols): CCL18 concentrations always below mean values. **(B)** Group 2 (blue and green symbols) Blue symbols: CCL18 concentrations equal or above mean values at T0 and or fluctuating CCL18 levels along the study. Green symbols: patients whose CCL18 effluent levels increased along the time of study.
(PDF)
