# Supplementary material for: Sustained low peritoneal effluent CCL18 levels are associated with preservation of peritoneal membrane function in peritoneal dialysis
Source: PLoS One. 2017 Apr 17;12(4):e0175835. doi: 10.1371/journal.pone.0175835 (PMC5393879; doi:10.1371/journal.pone.0175835)
Supplement: S1 Table — (PDF) [file pone.0175835.s001.pdf]

S1 Table. Demographic characteristics of 43 patients included in the longitudinal study.

	Baseline	1 year of PD	2 years of PD	3 years of PD
Mean Age	53 years (range 20–78 years)			
Sex (Men) N (%)	31 (68.88%			
Cause of Kidney failure	Chronic glomerulonephritis: 8 patients<br>Diabetic nephropathy (2 type I and 4 type II): 6 patients<br>Nephrosclerosis: 6 patients<br>Adult polycystic kidney disease: 5 patients<br>Obstructive uropathy: 5 patients<br>Systemic disease: 4 patients<br>Tubulointerstitial nephropathy: 2 patients<br>Undetermined: 7 patients			
Hypertension[1]	39 patients (92.9%)			
Diabetes mellitus	Type I: 2 patients (4.8%) // Type 2: 8 patients (19%)			
Dyslipidemia	29 patients (69%)			
Cardiac or cerebral stroke	13 patients (31%)			
Peripheral vascular disease	9 patients (21.4%)			
Obesity[2]	7 patients (16.7%)			
Major abdominal surgery	3 patients (7.1%)	3 patients (7.1%)	4 patients (9.8%)	4 patients (11.8%)
Steroid treatment	3 patients (7.1%)	4 patients (9.5%)	2 patients (4.9%)	1 patient (2.9%)
Tamoxifen treatment[3]	0	0	0	1 patient (2.9%)

[1]Blood preasure (BP)>140/90mmHg or specific treatment
[2]Body mass index (BMI)>30
[3]Used as prophylaxis for peritoneal fibrosis
