# Supplementary material for: Sustained low peritoneal effluent CCL18 levels are associated with preservation of peritoneal membrane function in peritoneal dialysis
Source: PLoS One. 2017 Apr 17;12(4):e0175835. doi: 10.1371/journal.pone.0175835 (PMC5393879; doi:10.1371/journal.pone.0175835)
Supplement: S2 Table — (PDF) [file pone.0175835.s002.pdf]

S2 Table. Demographic characteristics of 61 patients included in the cross-sectional study.

Mean Age	53 years (range 21–86 years)
Sex (men) N (%)	42 (68.85%)
Cause of Kidney failure	Chronic glomerulonephritis: 10 patients<br>Diabetic nephropathy (2 type I and 4 type II): 6 patients<br>Nephrosclerosis: 7 patients<br>Adult polycystic kidney disease: 6 patients<br>Obstructive uropathy: 8 patients<br>Systemic disease: 6 patients<br>Tubulointerstitial nephropathy: 2 patients<br>Undetermined: 16 patients
Hypertension[1]	52 patients (92.9%)
Diabetic mellitus	Type I: 2 patients (3.6%) // Type 2: 9 patients (16.1%)
Dyslipidemia	41 patients (73.2%)
Cardiac or cerebral stroke	21 patients (37.5%)
Peripheral vascular disease	20 patients (37.5%)
Obesity[2]	5 patients (8.9%)
Major abdominal surgery	7 patients (12.5%)
Steroid treatment	4 patients (7.4%)
Tamoxifen treatment[3]	3 patients (5.3%)

[1]Blood preasure (BP)>140/90mmHg or specific treatment
[2]Body mass index (BMI)>30
[3]Used as prophylaxis for peritoneal fibrosis
