# Supplementary material for: Sustained low peritoneal effluent CCL18 levels are associated with preservation of peritoneal membrane function in peritoneal dialysis
Source: PLoS One. 2017 Apr 17;12(4):e0175835. doi: 10.1371/journal.pone.0175835 (PMC5393879; doi:10.1371/journal.pone.0175835)
Supplement: S3 Table — (PDF) [file pone.0175835.s003.pdf]

**S3 Table. Effluent and serum CCL18 levels during a 3-year follow-up of longitudinal study.**

| **SERUM** CCL18 levels (ng/ml) | Baseline | 1 year of PD | 2 years of PD | 3 years of PD |
|---|---|---|---|---|
| MEDIAN± SD | 134.51 ± 66.51 | 149.59 ± 79.34 | 137.80 ± 71.41 | 151.59 ± 72.06 |
| **EFFLUENT** CCL18 levels (ng/ml) | Baseline | 1 year of PD | 2 years of PD | 3 years of PD |
| MEDIAN± SD | 2.82 ± 1.56 | 3.12 ± 2.05 | 2.94 ± 1.77 | 3.43 ± 1.78 |
