# Supplementary material for: Sustained low peritoneal effluent CCL18 levels are associated with preservation of peritoneal membrane function in peritoneal dialysis
Source: PLoS One. 2017 Apr 17;12(4):e0175835. doi: 10.1371/journal.pone.0175835 (PMC5393879; doi:10.1371/journal.pone.0175835)
Supplement: S4 Table — (PDF) [file pone.0175835.s004.pdf]

S4 Table. Demographic characteristics of patients from the longitudinal study included in Group 1 and Group 2

		Baseline	1 year of DP	2 years of DP	3 years of DP
Sex (men) N (%)	Group 1	7 (63.63%)			
	Group 2	24 (75%)			
Mean Age (years)	Group 1	54.81			
	Group 2	53.36			
Cause of kidney failure	Group 1	CGN 2 (18.2%) / DN 2 (18.2%) / APKD 1 (9.1%) / OU 1 (9.1%) / SD 1 (9.1%) / TN 1 (9.1%) / Undetermined 3 (27.3%)			
	Group 2	CGN 6 (19.4%) / DN 4 (12.9%) / APKD 4 (12.9%) / OU 4 (12.9%) / SD 3 (9.7%) / TN 1 (3.2%) / Undetermined 3 (9.7%)			
Hypertension	Group 1	11(100%)			
	Group 2	28 (90,3%)			
DM 1 / DM2	Group 1	DM1: 0 // DM2: 4 (36.4%)			
	Group 2	DM1: 2 (6.5) // DM2: 4 (12.9%)			
Dyslipidemia	Group 1	7 (63.3%)			
	Group 2	22 (71%)			
Cardiac or cerebral stroke	Group 1	3 (27.3%)			
	Group 2	10 (32.3%)			
Peripheral vascular disease	Group 1	2 (18.2%)			
	Group 2	7 (22.6%)			
Obesity	Group 1	1 (9.1%)			
	Group 2	6 (19.4%)			
Major abdominal surgery	Group 1	0	0	0	0
	Group 2	3 (9.7%)	3 (9.7%)	4 (13.3%)	4 (16%)
Steroid treatment	Group 1	1(9%)	1 (9%)	1 (9%)	0
	Group 2	2 (5.55%	3 (8.33)	1 (3.3%)	1 (4%)
Tamoxifen treatment	Group 1	0	0	0	0
	Group 2	0	0	0	1 (4%)

CGN: Chronic glomerulonephritis;
DN: Diabetic Nephropaty
APKD: Adult polycystic kidney disease
OU: Obstructive uropathy
SD: Systemic disease
TN: Tubulointeerstitial nephropathy
DM1: diabetes mellitus type1; DM2: diabetes mellitus type 2; DL: dislipidemia;
