# Supplementary material for: Sustained low peritoneal effluent CCL18 levels are associated with preservation of peritoneal membrane function in peritoneal dialysis
Source: PLoS One. 2017 Apr 17;12(4):e0175835. doi: 10.1371/journal.pone.0175835 (PMC5393879; doi:10.1371/journal.pone.0175835)
Supplement: S1 Fig — Time course analysis of serum (A) and effluent (B) CCL18 values in 43 patients treated with PD during a 3-year follow-up period. Box plots show median, interquartile ranges, and SD. No significant changes were found overall throughout PD (ANOVA test). (PDF) [file pone.0175835.s005.pdf]

S1 Figure

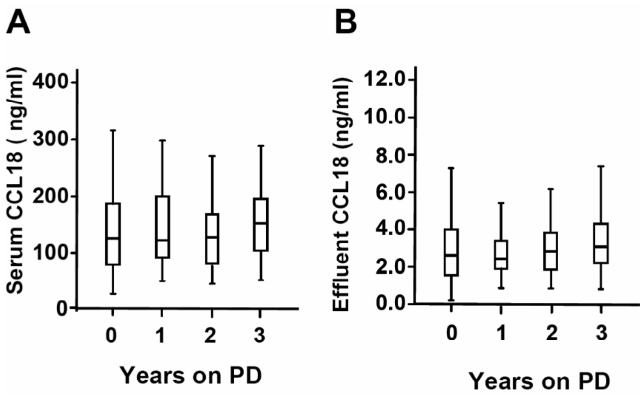

Supplemental Figure 1. Longitudinal study of CCL18 values in patients treated with PD. Time course analysis of serum **(A)** and effluent **(B)** CCL18 values in 43 patients treated with PD during a 3-year follow-up period. Box plots show median, interquartile ranges, and SD. No significant changes were found overall throughout PD (ANOVA test).
