# Supplementary material for: Sustained low peritoneal effluent CCL18 levels are associated with preservation of peritoneal membrane function in peritoneal dialysis
Source: PLoS One. 2017 Apr 17;12(4):e0175835. doi: 10.1371/journal.pone.0175835 (PMC5393879; doi:10.1371/journal.pone.0175835)
Supplement: S2 Fig — Effluent concentrations of CCL18 in patients included in group 1 and group 2 (A) Group 1(orange symbols): CCL18 concentrations always below mean values. (B) Group 2 (blue and green symbols) Blue symbols: CCL18 concentrations equal or above mean values at T0 and or fluctuating CCL18 levels along the study. Green symbols: patients whose CCL18 effluent levels increased along the time of study. (PDF) [file pone.0175835.s006.pdf]

S2 Figure

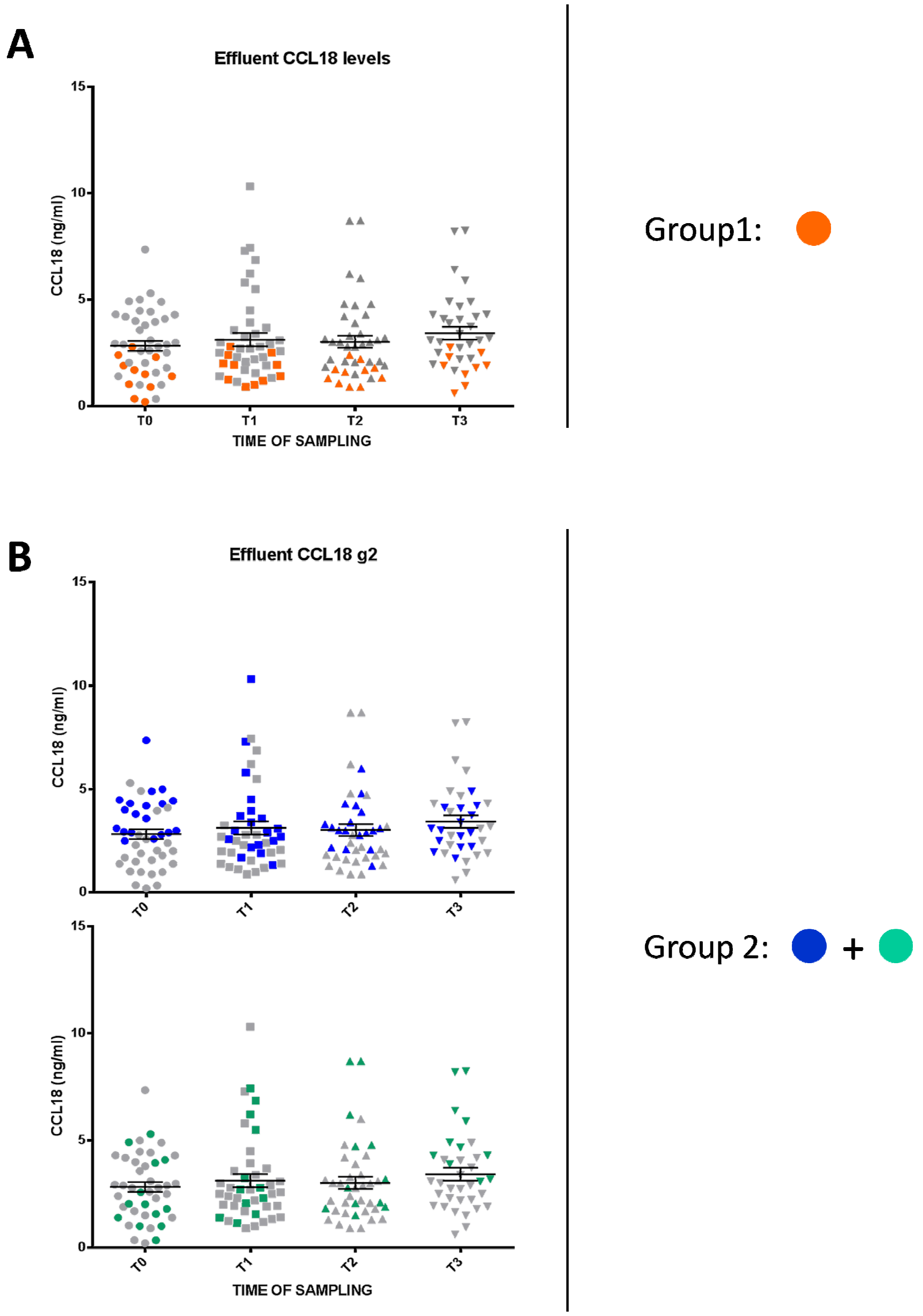

S2 Figure. Effluent concentrations of CCL18 in patients included in group 1 and group 2 (A) Group 1(orange symbols): CCL18 concentrations always below mean values. **(B) Group 2 (blue and green symbols)** Blue symbols: CCL18 concentrations equal or above mean values at T0 and or fluctuating CCL18 levels along the study. Green symbols: patients whose CCL18 effluent levels increased along the time of study**.**
